# Effects of tumor treating fields (TTFields) on human mesenchymal stromal cells

**DOI:** 10.1007/s11060-024-04740-0

**Published:** 2024-06-20

**Authors:** Maren Strack, Jan Kückelhaus, Martin Diebold, Patrick Wuchter, Peter E. Huber, Oliver Schnell, Roman Sankowski, Marco Prinz, Anca-Ligia Grosu, Dieter Henrik Heiland, Nils H. Nicolay, Alexander Rühle

**Affiliations:** 1 Department of Radiation Oncology, Medical Center, Faculty of Medicine, University of Freiburg, German Cancer Consortium (DKTK), partner site DKTK-Freiburg, Robert-Koch-Str. 3, 79106 Freiburg, Germany; 2 Department of Neurosurgery, Medical Center, Faculty of Medicine, University of Freiburg, Freiburg, Germany; 3 Institute of Neuropathology, Faculty of Medicine, University of Freiburg, Freiburg, Germany; 4 Neurology and Medical Oncology, University Hospital Basel and University of Basel, Basel, Switzerland; 5 Institute of Transfusion Medicine and Immunology, Medical Faculty Mannheim, German Red Cross Blood Service Baden- Württemberg– Hessen, Heidelberg University, Mannheim, Germany; 6 Department of Molecular Radiation Oncology, German Cancer Research Center (dkfz), Heidelberg, Germany; 7 Department of Radiation Oncology, University Hospital Center Heidelberg, Heidelberg, Germany; 8 Signalling Research Centres BIOSS and CIBSS, University of Freiburg, Freiburg, Germany; 9 Department of Radiation Oncology, University of Leipzig Medical Center, Leipzig, Germany; 10 Comprehensive Cancer Center Central (CCCG) Germany, Partner Site Leipzig, Leipzig, Germany

**Keywords:** Mesenchymal stem cells, Mesenchymal stromal cells, Glioma, Tumor microenvironment

## Abstract

**Purpose:**

Mesenchymal stromal cells (MSCs) within the glioblastoma microenvironment have been shown to promote tumor progression. Tumor Treating Fields (TTFields) are alternating electric fields with low intensity and intermediate frequency that exhibit anti-tumorigenic effects. While the effects of TTFields on glioblastoma cells have been studied previously, nothing is known about the influence of TTFields on MSCs.

**Methods:**

Single-cell RNA sequencing and immunofluorescence staining were employed to identify glioblastoma-associated MSCs in patient samples. Proliferation and clonogenic survival of human bone marrow-derived MSCs were assessed after TTFields in vitro. MSC’ characteristic surface marker expression was determined using flow cytometry, while multi-lineage differentiation potential was examined with immunohistochemistry. Apoptosis was quantified based on caspase-3 and annexin-V/7-AAD levels in flow cytometry, and senescence was assessed with ß-galactosidase staining. MSCs’ migratory potential was evaluated with Boyden chamber assays.

**Results:**

Single-cell RNA sequencing and immunofluorescence showed the presence of glioblastoma-associated MSCs in patient samples. TTFields significantly reduced proliferation and clonogenic survival of human bone marrow-derived MSCs by up to 60% and 90%, respectively. While the characteristic surface marker expression and differentiation capacity were intact after TTFields, treatment resulted in increased apoptosis and senescence. Furthermore, TTFields significantly reduced MSCs’ migratory capacity.

**Conclusion:**

We could demonstrate the presence of tumor-associated MSCs in glioblastoma patients, providing a rationale to study the impact of TTFields on MSCs. TTFields considerably increase apoptosis and senescence in MSCs, resulting in impaired survival and migration. The results provide a basis for further analyses on the role of MSCs in glioblastoma patients receiving TTFields.

**Supplementary Information:**

The online version contains supplementary material available at 10.1007/s11060-024-04740-0.

## Introduction

Mesenchymal stromal cells (MSCs) can be found in different tissues including the microenvironment of various cancers [1–3]. In glioblastoma, the number of glioma-infiltrating MSCs have been shown to inversely correlate with patient survival [3]. Glioblastoma-associated MSCs promote proliferation, invasiveness and angiogenesis of glioblastoma cells [4, 5], and mitochondrial transfer from MSCs to glioblastoma stem cells contributes to resistance against temozolomide [6]. The origin of glioblastoma-associated MSCs remains a matter of debate: differentiation from glioblastoma stem cells, epithelial-to-mesenchymal transition-like processes from astrocytes, transdifferentiation from pericytes and vascular smooth muscle cells, or migration of bone marrow-MSCs towards glioblastomas have been discussed [7].

Tumor Treating Fields (TTFields) are alternating electric fields with low intensity (1–3 V/cm) and intermediate frequency (100–500 kHz) that can disrupt the localization and orientation of polar molecules such as tubulin and septin, finally impairing key processes during mitosis [8, 9]. Besides these antimitotic effects, TTFields result in alterations of several biological processes including DNA repair, autophagy and migration [10–13], summarized by Karanam and colleagues [14]. In the last years, it could be shown that TTFields cause immunogenic cell death and exhibit an immunoactivating role [15–17]. TTFields not only target tumor cells themselves but also influence the tumor microenvironment and permeabilize the blood-brain barrier [18–20]. In accordance with the preclinical data, TTFields have demonstrated clinical efficacy in the treatment of glioblastoma, pleura mesothelioma and non-small-cell lung cancer [21–24].

Given the pro-tumorigenic effects of glioblastoma-associated MSCs, we aimed to examine the role of MSCs in glioblastoma patients and to explore the influence of TTFields on MSCs in vitro.

## Methods

### Single-cell RNA sequencing

For single-cell analysis, we used the GBMap reference dataset [25, 26]. Single-cell data were processed using the Seurat (v5.0) package in R software. To identify the MSC population within the single cell data, we first filtered the cells by the expression of classical positive marker genes for MSCs (*NT5E*, *THY1*, *ENG*, and *ITGB1*) [27]. We built a meta score using the Seurat function *Seurat::AddModuleScore()*. Next, we removed all cells containing high expression (> 50% quantile) of MSC-negative marker genes (*CD14*, *CD34*, *PTPRC*) and subsequently, all cells bearing aneuploid chromosomal alterations (based on the *inferCNV [copy number variations] estimations*) to avoid contamination with tumor cells.

### Immunofluorescence staining

In a cohort of 11 glioblastoma patients, all treated with surgical resection, (chemo)radiation and TTFields treatment, the expression of the MSC markers CD73, CD90 and CD105 was analyzed at initial diagnosis. Formalin-fixed paraffin-embedded tissue sections of 4 μm thickness were mounted on glass slides for deparaffinization and heat-induced antigen retrieval at pH 9, subsequent blocking, indirect immunofluorescence staining (Supplementary Table 1) and DAPI staining (Carl Roth, Karlsruhe, Germany). Randomly selected representative images from the lesion area were acquired on a Keyence BZ-X810 fluorescence microscope (Keyence Corporation, Osaka, Japan) in quadruplets. Total cell numbers were determined using the particle analyzation plug-in for ImageJ (National Institutes of Health, Bethesda, USA). Cells larger than 10 μm and equally expressing CD73, CD90 and CD105 were counted.

### Cell culture

Human MSCs were isolated from the bone marrow of three healthy donors and cultured in StemMACS™ human MSC Expansion Medium (Miltenyi Biotec, Bergisch-Gladbach, Germany), supplemented with 1% (v/v) penicillin/streptomycin [28, 29]. Written consent was obtained before bone marrow sampling, and the investigation was approved in advance by the Heidelberg University Ethics Committee (S-384/2004) and the Freiburg University Ethics Committee (436/20).

In order to compare the results obtained with MSCs, HS68 (RRID: CVCL_0839) fibroblasts were used as control cell population. HS68 fibroblasts were purchased from the American Type Culture Collection (ATCC, Manassas, VA, USA) and cultured in Dulbecco’s Modified Eagle Medium (DMEM), containing 1 g/L glucose and GlutaMAX™ (Gibco, Carlsbad, USA), supplemented with 10% fetal bovine serum (FCS) and 1% penicillin/streptomycin.

All cells were maintained at 37 °C in a humidified incubator with 5% CO_2_, and the medium was changed twice a week.

### TTFields

TTFields were applied using the inovitro™ system (Novocure, Haifa, Israel) [30]. Cells were exposed to the electric fields for 72 h prior to analyses. 2 × 10^4^ cells were plated on cover slips and left to adhere overnight prior to TTFields application. For proliferation and viability analyses, TTFields were applied at an intermediate (~ 1,33 V/cm root-mean-square (RMS)) and a high (~ 1,62 V/cm RMS) field intensity, and several frequencies within the range of 100–500 kHz were used. For all other endpoints, cells were treated at an intensity of 1.33 V/cm and the clinically applied frequency of 200 kHz.

### Proliferation, clonogenic survival, and viability

After TTFields treatment, cells were detached using trypsin and counted using a Neubauer chamber. Trypan blue staining was performed to select viable cells.

For clonogenic survival analyses, cells were treated with TTFields and then replated in T25 cell culture flasks. Cells were allowed to divide for 10–14 days before fixation and staining using a methanol/crystal violet solution, as described before [31]. Colonies with ≥ 50 cells were counted with a light microscope. Cellular survival fractions were calculated using the formula (no. of colonies/ no. of cells plated)_treated_/(no. of colonies/ no. of cells plated)_untreated_.

Cellular viability was determined using resazurin assays. After TTFields treatment, 2 × 10^3^ cells, suspended in 100 µL of the respective cell culture medium, were replated in a 96-well plate. After 96 h, 10 µL of 0.3 mg/mL resazurin (PromoCell, Heidelberg, Germany) was added to each well and incubated for 4 h. Colorimetric analyses were performed by measuring light absorbance at 570 nm and 600 nm using a VersaMax microplate reader (Molecular Devices, LLC, Sunnyvale, CA, USA). To quantify cellular capacity to metabolize resazurin, background absorbance at 600 nm was subtracted from absorbance at 570 nm.

### Cell cycle and apoptosis

Cells were fixed in a 3% paraformaldehyde (PFA)/phosphate-buffered saline (PBS) solution immediately after TTFields treatment and permeabilized using − 20 °C-cold 70% ethanol. After blocking with 0.5% bovine serum albumin (BSA) in PBS, samples were incubated with an Alexa Fluor 647-conjugated antibody targeting cleaved caspase-3 (BD Pharmingen, Heidelberg, Germany), diluted 1:20 in 3% BSA/PBS, for 1 h at room temperature. After centrifugation and removal of the supernatant, 250 µL 4′,6-diamidino-2-phenylindole (DAPI)/PBS solution (1 µg/mL) was added to analyze the cell cycle distribution.

To further differentiate between early and late apoptosis, annexin V/7-aminoactinomycin D (7-ADD) measurements were performed. Viable MSCs and fibroblasts were harvested after being exposed to TTFields for 72 h and stained with the PE-Annexin-V Apoptosis Detection Kit with 7-AAD (BioLegend, London, UK) following the manufacturer’s protocol.

Flow cytometry measurements were performed using a FACSVerse™ (BD Biosciences, San Jose, CA, USA) and quantified using FlowJo™ v10 (FlowJo LLC).

### Senescence

2 × 10^3^ were plated on glass cover slips and exposed to TTFields for 72 h, before cover slips were transferred to a 24-well plate to be fixed and stained using the Senescence β-Galactosidase Staining Kit (Cell Signaling Technology, Danvers, MA, USA). Nuclei were counterstained with 1 µg/mL DAPI/PBS. Five randomly chosen regions of interest (ROI) per technical replicate were photographed using an Olympus IX51 microscope (Olympus Corporation, Tokyo, Japan). Senescent cells were manually counted in each ROI, while the total number of cells were automatically determined using ImageJ.

### Cell cytoskeleton

As TTFields are known to alter the cytoskeletal structure of glioblastoma cells, thereby impairing the migratory potential, we performed F-actin immunofluorescence staining to examine the influence of TTFields on the cytoskeleton of MSCs. After TTFields treatment, 1 × 10^3^ cells were plated on glass cover slips in 24-well plates. Cells were fixed using a 3% PFA/PBS solution, permeabilized with 70% ethanol at -20 °C for 30 min, and stained with AF488-conjugated phalloidin (Invitrogen, Waltham, MA, USA), diluted in 1% BSA/PBS, for 60 min at room temperature. Cell nuclei were counterstained with 1 µg/mL DAPI/PBS.

### Migration

Boyden chamber migration assays based on an FCS-gradient were performed to examine the effect of TTFields on MSCs’ migratory potential. To sensitize the cells to the gradient, MSCs and fibroblasts were cultured in FCS-free DMEM for 48 h. 2 × 10^3^ FCS-starved cells were then pipetted into a transwell insert (24 well ThinCert-TC Inserts, pore size 8 μm, Greiner Bio-One, Frickenhausen, Germany) and transferred to inovitro™ high wall dishes containing DMEM supplemented with 10% FCS (for MSCs) and 20% FCS (for HS68). Afterwards, high wall dishes were connected to TTFields base plates to initiate the treatment. After 24-hours TTFields treatment, cells that had not migrated were cleared off the upper membrane surface. Transwells were washed in PBS and placed in a 4% PFA/PBS solution for 15 min. Nuclei were stained with 1 µg/mL DAPI/PBS, and images of 4 ROIs per transwell were taken. Total cell number in these sections was determined using the particle analyzation plug-in for ImageJ.

### Adhesion

TTFields-associated increase of cellular adhesion was investigated as previously described [13]. The average time needed for detachment of untreated cells was determined for each cell line individually. Subsequently, TTFields-treated cells were incubated in trypsin for exactly this period of time, and cells detached after this time span were counted using a Neubauer chamber. To compensate for generally lower cell numbers in TTFields-treated specimens, a second trypsinization step was performed until all treated cells were detached, and cells were counted again. The detachment rate was calculated by the formula (No. of cells detached)_1st trypsinization_/((No. of cells detached)_1st trypsinization_+(No. of cells detached)_2nd trypsinization_).

### Surface marker expression

To examine whether TTFields affect the expression of the defining MSC surface marker pattern [27], flow cytometric measurements were performed as reported before [32]. After harvesting, specimens were washed and resuspended in FACS buffer containing 0.5% BSA/2 mM ethylenediaminetetraacetic acid in PBS. 10 µL of MSC Phenotyping solution (MSC Phenotyping Kit, human, Miltenyi Biotec) was added, followed by incubation for 10 min. MSC markers were targeted using anti-CD73-APC, anti-CD90-FITC and anti-CD105-PE antibodies, whereas PerCP-conjugated anti-CD14/CD20/CD34/CD45-antibodies were used regarding MSC negative markers. Fluorescence signatures were measured on a FACSVerse™ and analyzed with FlowJo™ v10.

### MSC differentiation

After 72-hour TTFields treatment, cells were seeded for subsequent differentiation experiments. For adipogenic and osteogenic differentiation, 3 × 10^4^ cells per well were plated in 24-well plates. StemMACS™ AdipoDiff Media (Miltenyi Biotec) was used to induce adipogenic differentiation, while osteogenic differentiation was induced by StemMACS™ OsteoDiff Media (Miltenyi Biotec). Lipid droplets of MSC-derived adipocytes were stained using 1 µM/mL BODIPY® 493/503, whereas hydroxyapatite secreted by MSC-derived osteocytes was stained with OsteoImage^™^ Staining Reagent (Lonza, Basel, Switzerland).

To analyze chondrogenic differentiation, 1 × 10^5^ MSCs were transferred to each well of 96-well plates and allowed to form spheroids. Chondrogenic differentiation was included with StemMACS^™^ ChondroDiff Media (Miltenyi Biotech). After 21 days, spheroids were fixed with 4% PFA/PBS solution for 30 min, frozen at -20 °C and sectioned using a cryomicrotome. Sections were incubated in 1% alcian blue dissolved in 3% acetic acid solution for 30 min, and washed with 0.1 M hydrochloric acid, PBS and deionized water.

### Statistics

At least three replicates were used for all experiments. Values are presented as mean with standard deviations. Potential differences between the TTFields and control group were examined using unpaired t-tests. Statistical analyses and visualization of the results were performed using GraphPad Prism 9 (GraphPad Software Inc., San Diego, CA, USA). A *p*-value < 0.05 was considered as statistically significant.

## Results

### Abundance of MSCs in glioblastoma

**Fig. 1:**
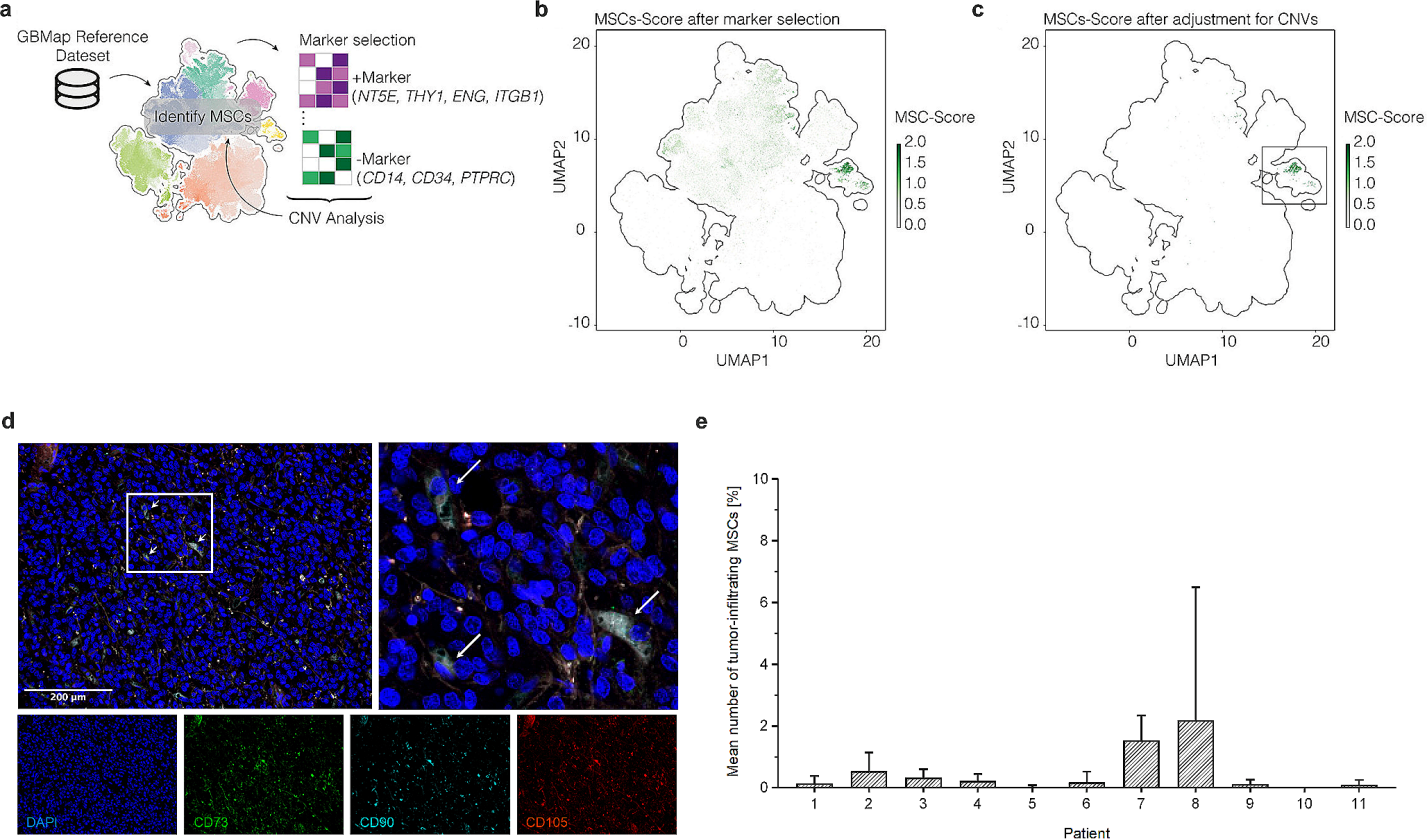
Single-cell RNA sequencing and immunofluorescence reveal MSCs within the glioblastoma microenvironment. (**a**) Illustration of the workflow regarding identification of glioblastoma-associated MSCs. (**b**) Scatter plot of the dimensional reduction (UMAP) of the GBMap single cell dataset. Colors indicate the MSCs-score after filtering for positive and negative marker. (**c**) UMAP representation of the MSC cells after removal of cells containing chromosomal alterations. (**d**) Representative immunofluorescence staining images of CD73/CD90/CD105-positive cells (considered as MSCs). Blue: DAPI-stained nuclei, green: CD73, cyan: CD90, red: CD105, white: CD73/CD90/CD105-positive cells. Arrows: exemplary triple-positive cells classified as MSCs. Scale bar, 200 μm. (**e**) Percentage of CD73/CD90/CD105-positive cells (considered as tumor-infiltrating MSCs) in 11 patients with glioblastoma undergoing surgery, (chemo)radiation and subsequent TTFields treatment. Stainings were performed on the initial specimen at primary surgery

We accomplished MSC identification through single-cell RNA-sequencing and a dual marker strategy, utilizing positive (NT5E, THY1, ENG, ITGB1) and negative markers (CD14, CD34, PTPRC), followed by the exclusion of cells with aneuploid chromosomal alterations (Fig. 1a-c). After demonstrating the presence of these multipotent stromal cells within the glioblastoma microenvironment, we conducted immunofluorescence analyses of CD73, CD90 and CD105 in glioblastoma patient samples at initial surgery in order to validate these findings (Fig. 1d-e, Supplementary Table 2). Here, CD73/CD90/CD105-positive cells were found in 10 out of the 11 analyzed patients, with a mean abundance value of 0.5% (range, 0.0-2.2%).

### TTFields reduce MSCs’ proliferation and clonogenic survival

**Fig. 2:**
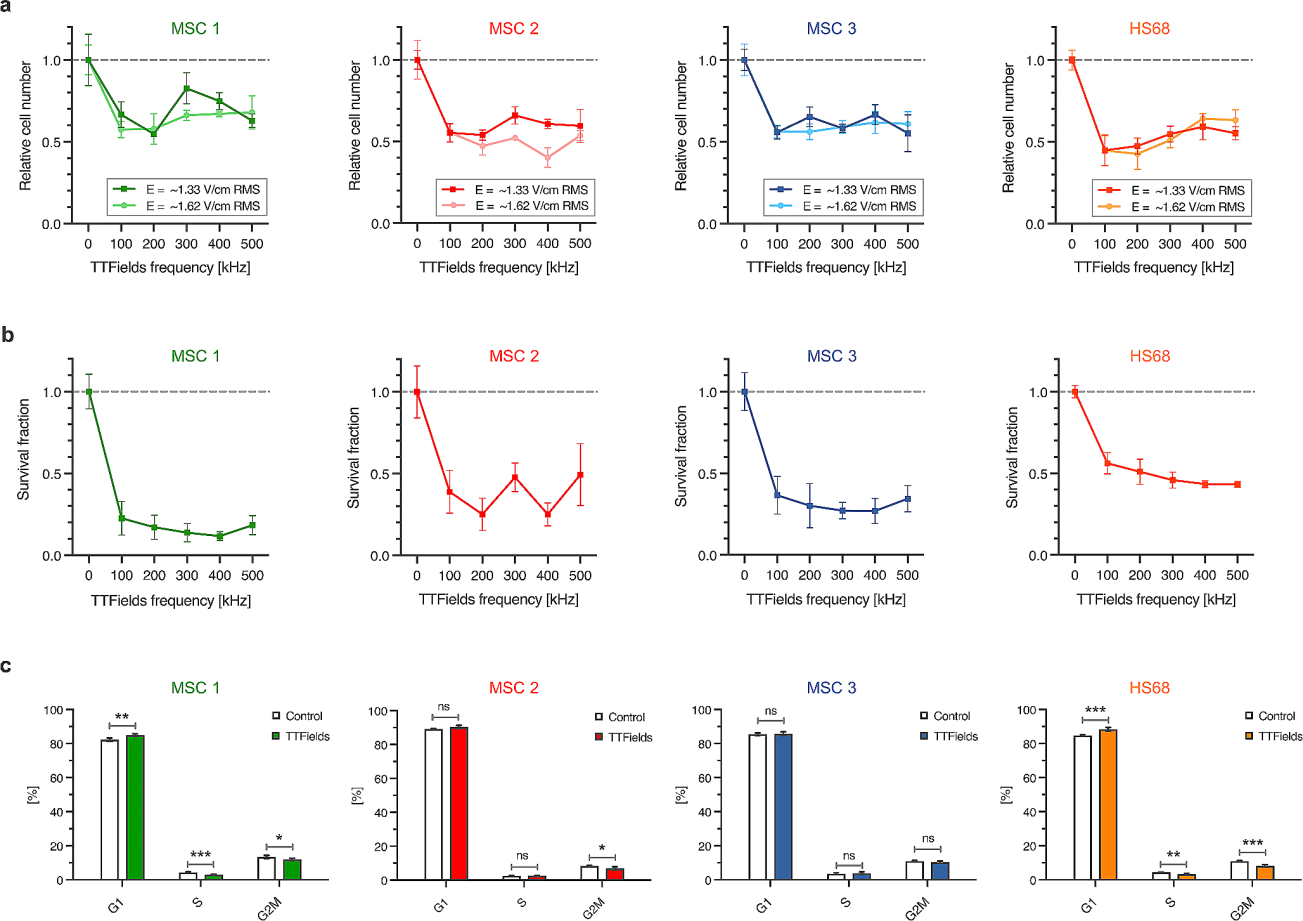
TTFields result in reduced proliferation and clonogenic survival in human MSCs. (**a**) Relative number of viable cells after 72 h of TTFields treatment depending on the electric field frequency in different MSCs and HS68 fibroblasts. Two different intensities (1.33 V/cm RMS and 1.62 V/cm RMS) were used. (**b**) Clonogenic survival of MSCs and HS68 fibroblasts after TTFields with an intensity of 1.33 V/cm using different electric field frequencies. (**c**) Cell cycle distribution of MSCs and HS68 fibroblasts after TTFields exposure at an intensity of 1.33 V/cm and a frequency of 200 kHz. Mean numbers (± standard deviations) are shown. **p < 0.01; ***p < 0.001; n.s, not significant. RMS, root-mean-square

In vitro, TTFields reduced the proliferation rates of human bone marrow-derived MSCs by about 50% (Fig. 2a). While there was a frequency optimum at 100–200 kHz in HS68 fibroblasts, no frequency optimum could be found for MSCs. Proliferation curves showed no significant differences between the two tested field intensities in MSCs (*p* = 0.27 for MSC1, *p* = 0.06 for MSC2, *p* = 0.60 for MSC3, paired t-tests) and HS68 fibroblasts (*p* = 0.73).

MSCs exhibited reduced clonogenic survival rates after TTFields by up to 88.3% in MSC1, 75.0% in MSC2 and 73.1% in MSC3 (Fig. 2b). At 200 kHz, which is clinically used in glioblastoma treatment [33], HS68 fibroblasts exhibited higher survival rates (50.9%) than MSCs (17.1% in MSC1, 25.0% in MSC2, 30.2% in MSC3). Metabolic viability of both MSCs and HS68 was not markedly reduced following TTFields exposure (Supplementary Material).

After TTFields treatment for 72 h, treatment effects on the cell cycle distribution of MSCs were small. While TTFields resulted in a minor G1 phase arrest in MSC1 cells (*p* < 0.01), there was no G1 phase arrest in MSC2 and MSC3 cells (Fig. 2C).

### TTFields induce apoptosis and senescence in MSCs

**Fig. 3:**
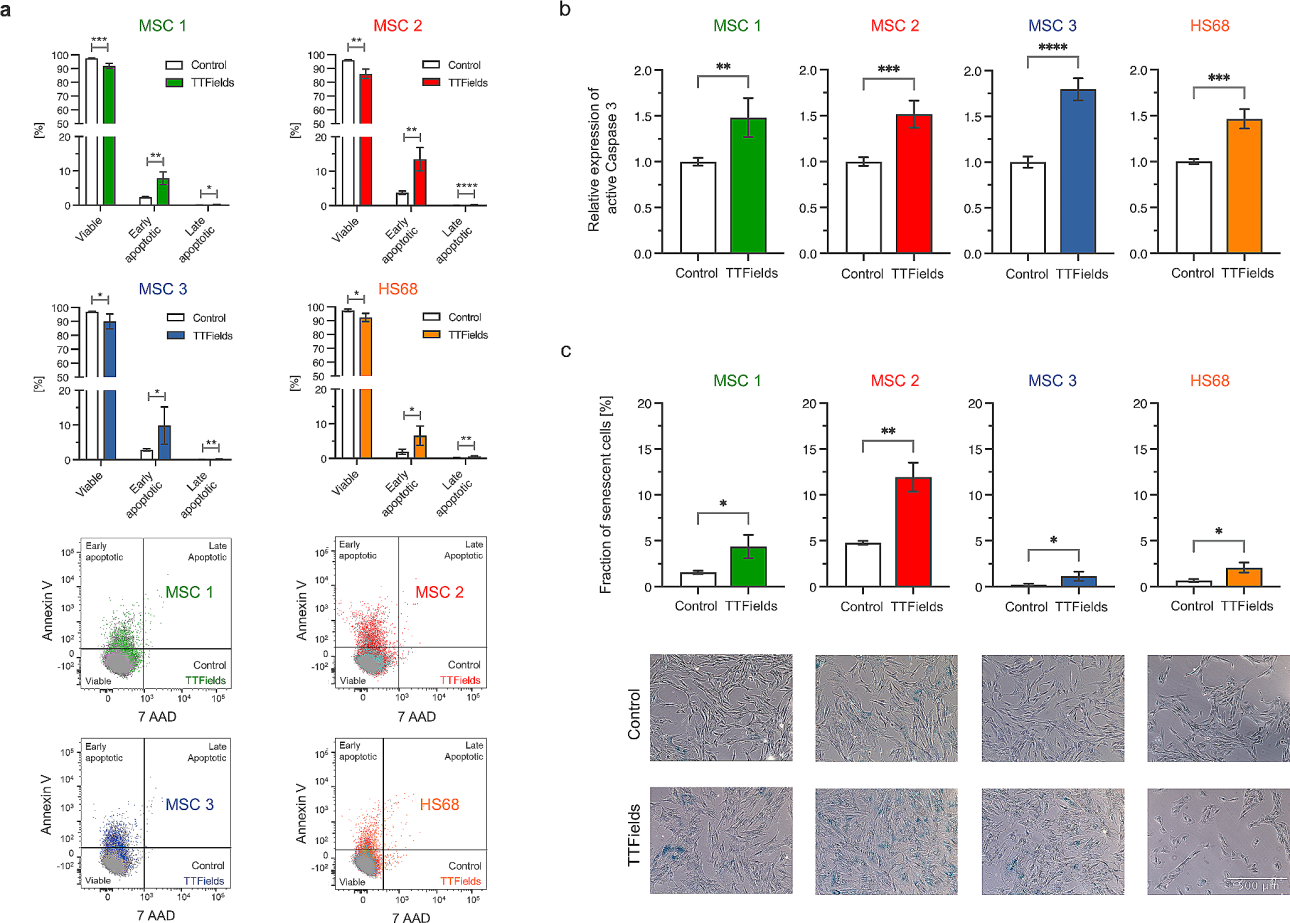
TTFields lead to increased apoptosis and senescence in human MSCs. (**a**) Percentage of early and late apoptotic cells after TTFields as assessed by annexin-V/7-AAD flow cytometry analyses. Representative scatter plots show the shift towards more annexin-V- and 7-AAD-positive cells after TTFields treatment compared with untreated controls. (**b**) Relative expression of cleaved caspase-3 in TTFields-treated cells in relation to untreated control cells as examined using flow cytometry measurements. (**c**) Percentage of β-galactosidase-positive cells after TTFields. Representative images of β-galactosidase staining are shown. Scale bar, 500 μm. Mean numbers with the standard deviations are shown. *p < 0.05; **p < 0.01; ***p < 0.001; ****p < 0.0001

Annexin-V/7-AAD assays revealed a TTFields-induced increase of annexin V-positive MSCs (*p* < 0.05 for MSC1 and MSC2, *p* < 0.01 for MSC3) (Fig. 3a). While untreated controls exhibited between 2.4% (MSC3) and 3.8% (MSC2) annexin-V-positive cells, TTFields led to apoptosis rates between 8.0% (MSC3) and 13.7% (MSC2). Apoptosis levels based on cleaved caspase-3 expression also showed elevated apoptosis rates after TTFields in MSC1 (*p* < 0.01), MSC2 (*p* < 0.001) and MSC3 (*p* < 0.0001) (Fig. 3b). Furthermore, the number of ß-galactosidase-positive cells was higher in all MSCs (*p* < 0.05 for MSC1 and MSC3, *p* < 0.01 for MSC2) following 72 h of TTFields treatment (Fig. 3c). The strongest increase was observed in MSC2 in which senescence levels doubled from 4.8 to 11.9%.

### TTFields impair MSCs’ migratory potential

**Fig. 4:**
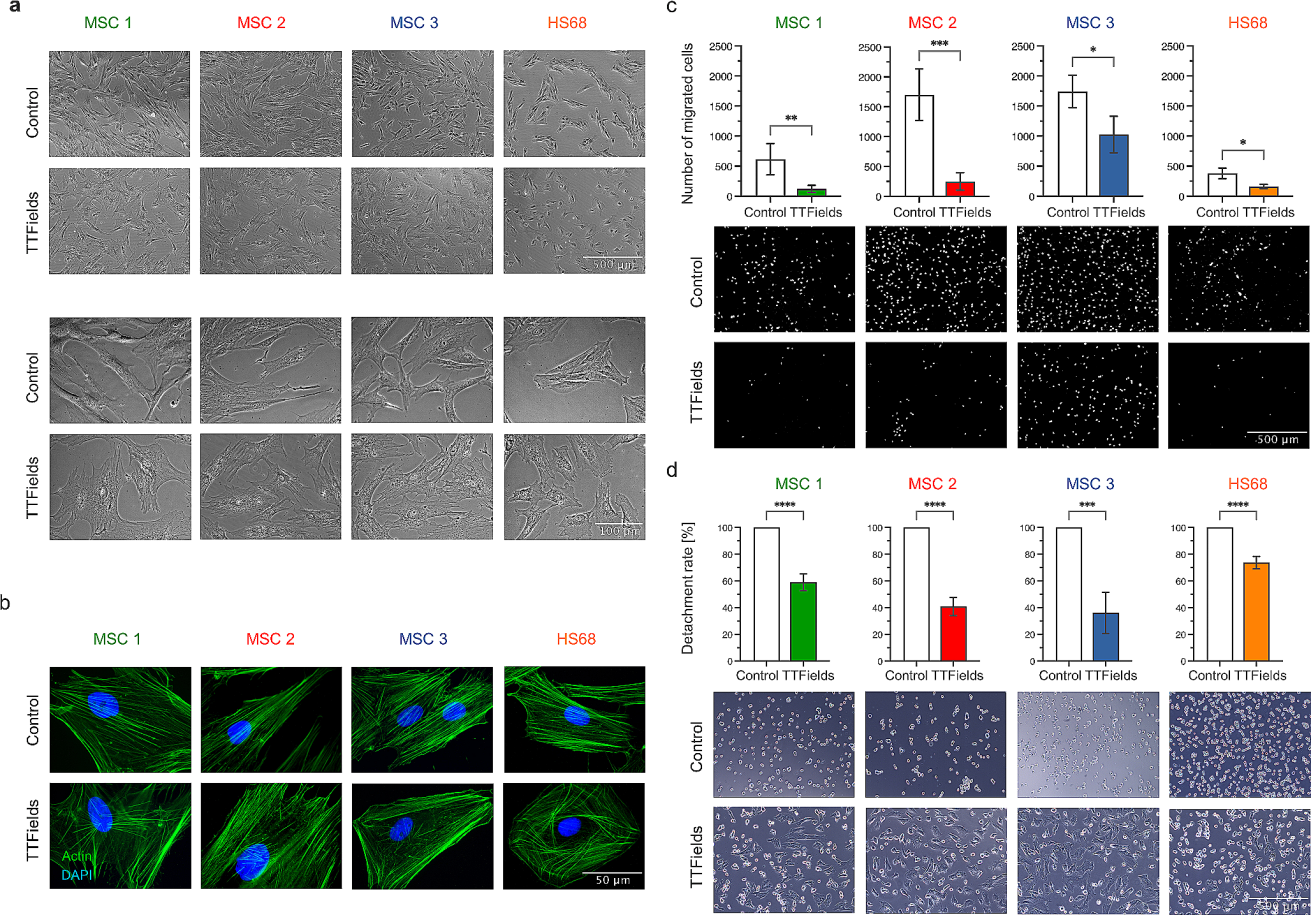
TTFields alter MSCs’ actin cytoskeleton and reduce MSCs’ migratory potential. (**a**) Representative phase contrast images of TTFields-exposed and untreated cells. Scale bars, 500 μm and 100 μm. (**b**) Immunofluorescence F-actin staining demonstrate cytoskeleton reorganization of TTFields-treated MSCs. Scale bar, 50 μm. (**c**) Number of migrating cells after TTFields as examined with classic Boyden chamber assays in 4 regions of interests (ROIs). Scale bar, 500 μm. (**d**) Detachment rate as surrogate marker for cellular adhesion ability is shown in control and TTFields-treated cells. The detachment rate was calculated by the formula (No. of cells detached)_1st trypsinization_/((No. of cells detached)_1st trypsinization_+(No. of cells detached)_2nd trypsinization_). Representative images show higher numbers of adherent cells after trypsinization in TTFields-treated cells. Scale bar, 500 μm. Mean values (± standard deviation) are shown. *p < 0.05; **p < 0.01; ***p < 0.001; ****p < 0.0001

TTFields-exposed MSCs were enlarged and exhibited more cytoplasmic vacuoles, supporting the finding of increased senescence induction after TTFields (Fig. 4a). F-actin immunofluorescence staining revealed a dense meshwork of actin filaments around the entire cell periphery in TTFields-treated MSCs (Fig. 4b). The number of migrated MSCs considerably decreased after TTFields exposure (*p* < 0.01 for MSC1, *p* < 0.001 for MSC2, *p* < 0.05 for MSC3) (Fig. 4c).

The trypsinization time, used as surrogate parameter for cellular adhesion, increased threefold in all MSCs (*p* < 0.0001 for MSC1, *p* < 0.001 for MSC2, *p* < 0.01 for MSC3) (Fig. 4d, Supplementary Material).

### MSCs’ stem cell characteristics remain unaltered after TTFields

**Fig. 5:**
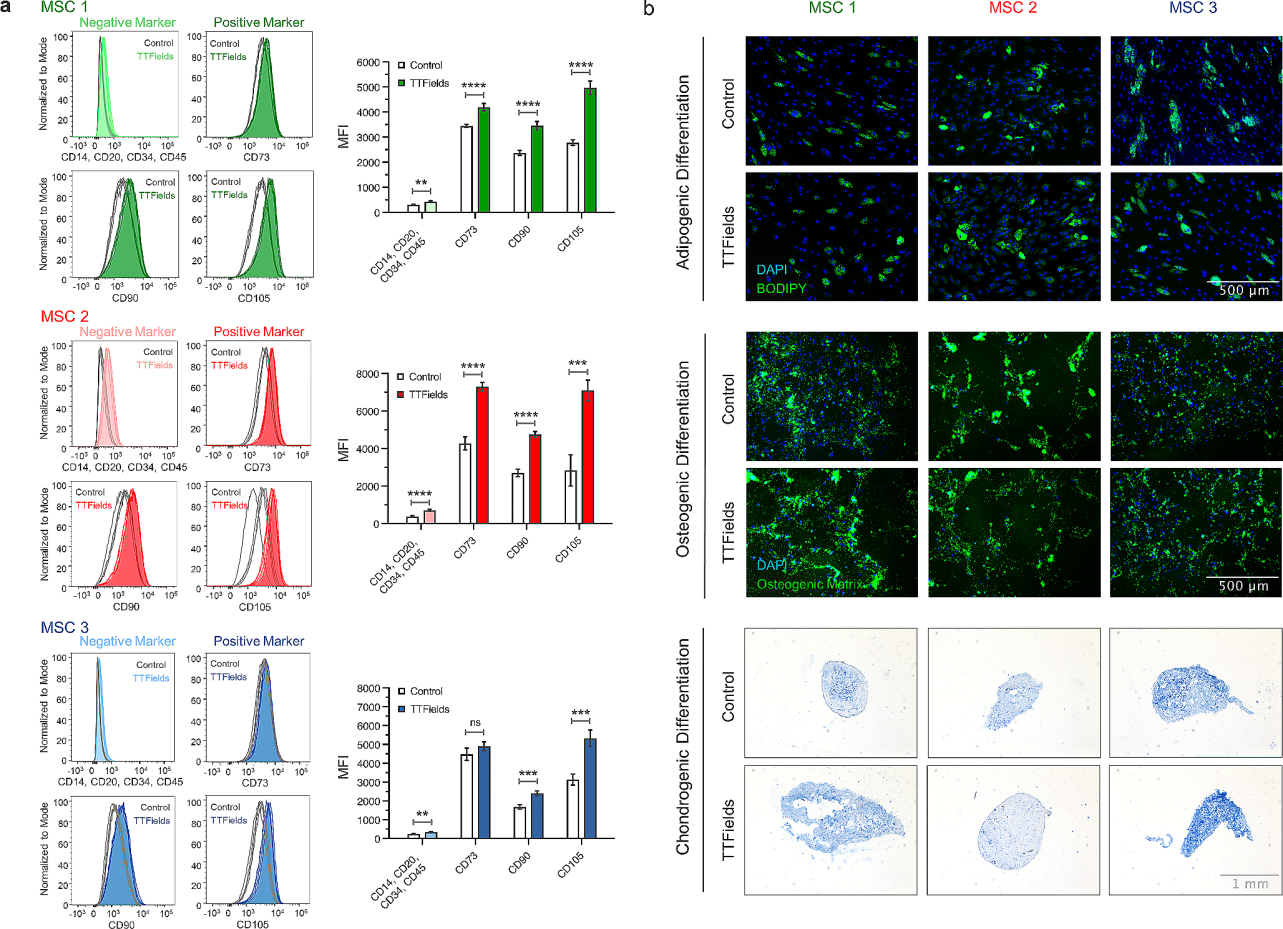
MSCs maintain their stem cell characteristics after TTFields. (**a**) Representative histograms of flow cytometry staining for the characteristic surface marker pattern of MSCs are shown, and mean fluorescence intensity (MFI) values of both MSC-negative (CD14, CD20, CD34, CD45) and MSC-positive marker (CD73, CD90, CD105) are presented. (**b**) Representative immunohistochemical images of adipogenic, osteogenic and chondrogenic differentiation of MSCs after TTFields. BODIPY® 493/503, OsteoImage^™^, and alcian blue stainings were performed regarding adipogenic, osteogenic, and chondrogenic differentiation, respectively. Mean values (± standard deviation) are shown. Scale bar, 500 μm for adipogenic und osteogenic differentiation, and 1 mm for chondrogenic differentiation. **p < 0.01; ***p < 0.001; ****p < 0.0001; n.s, not significant

All three investigated MSC samples were positive for CD73, CD90 and CD105. TTFields exposure for 72 h led to higher expression of these surface markers in MSC1-3 (Fig. 5a). As observed in flow cytometry analyses, cells were significantly larger after TTFields exposure, potentially contributing to the increased surface marker expression (Supplementary Material). As examined with immunohistochemical staining, exposure to TTFields did not mitigate the potential of MSCs to differentiate into adipocytes, osteocytes, and chondrocytes (Fig. 5b).

## Discussion

In this comprehensive preclinical and translational study, we could demonstrate the presence of MSCs within the glioblastoma microenvironment. Preclinically, human MSCs were found relatively sensitive towards TTFields. Their migratory potential and adhesive abilities were hampered, potentially related to alterations in the MSC actin cytoskeleton caused by TTFields. Both apoptosis and senescence levels were found to be increased after TTFields in MSCs, whereas the differentiation ability and surface marker expression of MSCs were unaltered.

To the best of our knowledge, this is the first study in which the effects of TTFields on human MSCs were studied. Both single-cell RNA sequencing and immunofluorescence analyses identified CD73/CD90/CD105-positive cells in the glioblastoma microenvironment, providing a rationale to study the influence of TTFields on MSCs [3, 4, 34, 35]. The majority of previous studies about TTFields focused on different cancer cells, while few studies examined TTFields’ effects on normal tissue cells such as astrocytes and neurons [36]. Previous studies could show that TTFields result in immunogenic cell death and furthermore induce STING and AIM2 inflammasome activation, thereby activating the adaptive immunity [15, 16]. As MSCs have been reported to exhibit immunosuppressive functions in the glioblastoma microenvironment, the anti-proliferative effects of TTFields on MSCs may further enhance the immunogenic potential of TTFields [37].

The migration of MSCs towards glioblastoma potentially contributes to the pro-tumorigenic ability of MSCs in the glioblastoma microenvironment [38, 39]. In our analysis, TTFields considerably impaired the migration capability of MSCs. A reduced migratory capacity after TTFields has also been described for other cell types, including glioblastoma, osteosarcoma, and liposarcoma cells [13, 40, 41]. It has been demonstrated in a previous study that TTFields alter the organization and dynamics of microtubules and actin, thereby hampering both cellular adhesion and migration [13]. These findings provide a preclinical rationale that TTFields also impair tumor cell metastatic activity, as observed in an in vivo study by Kirson and colleagues [18]. Considering the fact that MSCs exhibit a strong tropism towards glioblastoma cells, one may hypothesize that the reduced migration ability of MSCs after TTFields could negatively impact MSCs’ glioblastoma tropism [38].

In contrast to several cancer cells, MSCs did not exhibit a clear TTFields frequency optimum [12, 42, 43]. It has been shown that the optimal frequency is dependent on cellular morphology and size; Kirson et al. observed an inverse relationship between cellular size and the optimal TTFields frequency [42]. A previous preclinical investigation in which the effects of TTFields on head-and-neck squamous cell carcinoma cells were examined, did not show a distinct frequency optimum in terms of maximal reduction of cell proliferation either [44]. Although there was no clear frequency optimum, TTFields treatment with 200 kHz, as used for glioblastoma treatment in clinical settings, resulted in considerably reduced proliferation rates (about 50%) and clonogenic survival (80–90%) in MSCs.

A limitation of our study is the fact that we used bone marrow-derived MSCs instead of glioblastoma-derived MSCs. Bone marrow-derived MSCs have been discussed as potential source of glioblastoma-associated MSCs due to MSCs’ strong glioma tropism, and there are some similarities described between the bone marrow and the glioblastoma stem cell niche [45–47]. However, further studies are required to examine the effects of TTFields on glioblastoma-derived MSCs.

In conclusion, we could show for the first time that TTFields considerably impair survival and migratory capacity of human MSCs. Our findings provide a basis for further analyses on the role of MSCs in glioblastoma patients undergoing TTFields treatment. Our results may also have implications for other cancer types in which TTFields are currently investigated, as tumor-associated MSCs are also found in the stromal compartment of many cancer entities [48].

### Electronic supplementary material

Below is the link to the electronic supplementary material.

Supplementary Material 1

## Data Availability

The data that support the findings of this study are available from the corresponding author upon reasonable request.
