# Supplementary material for: Effects of tumor treating fields (TTFields) on human mesenchymal stromal cells
Source: J Neurooncol. 2024 Jun 20;169(2):329–40. doi: 10.1007/s11060-024-04740-0 (PMC11341748; doi:10.1007/s11060-024-04740-0)

Supplementary Data of

Effects of tumor treating fields (TTFields) on human mesenchymal stromal cells

Supplementary Tables

Supplementary Tab. 1. Details of primary antibodies used for immunohistochemistry stainings of glioblastoma specimens.

Channel	Epitope	Antibody	Clone	Concentration	Host
**647 nm**	CD105	R&D Human Endoglin/CD105 Antibody	NA (lot: AF1097)	5 µg/mL	Goat
**568 nm**	CD90	Sigma Monoclonal Anti-THY1 antibody	CL1028 (lot: MAB-02749)	1:50	Mouse
**488 nm**	CD73	Sigma Anti-NT5E antibody	NA (lot: 000003206)	1:50	Rabbit

Supplementary Tab. 2. Patient characteristics of glioblastoma patients (n=11) undergoing primary surgery, adjuvant (chemo)radiation and subsequent TTFields treatment. IQR, interquartile range; TMZ, temozolomide.

Characteristic	Number
**Age, median (IQR), years**	62 (55-63.5)
**Sex**	
**Female**	2
**Male**	9
**Chemoradiation regimen**	
**Chemoradiation with TMZ, 60 Gy in 30 fractions**	8
**Chemoradiation with TMZ, 40.05 Gy in 15 fractions**	1
**Chemoradiation with TMZ and lomustine, 60 Gy in 30 fractions**	1
**Radiotherapy alone, 60 Gy in 30 fractions**	1
**Radiotherapy completion**	
**Radiotherapy completed**	11
**Radiotherapy not completed**	0

Supplementary figures

(**A**) The relative viability of MSCs and fibroblasts after TTFields treatment determined by a resazurine assay is shown for different TTFields intensities and frequencies. Mean numbers with the according standard deviations are shown. (**B**) Required time of trypsin exposure up to total detachment of all cells was analyzed for MSCs and HS68 fibroblasts. Mean numbers with the according standard deviations are shown. **p*<0.05; ***p*<0.01; ****p*<0.001; *****p*<0.0001. (**C**) Representative FSC-A and SSC-A dot plots for naïve and TTFields-treated cells as determined by flow cytometry showing an increase in the cell size after TTFields treatment.

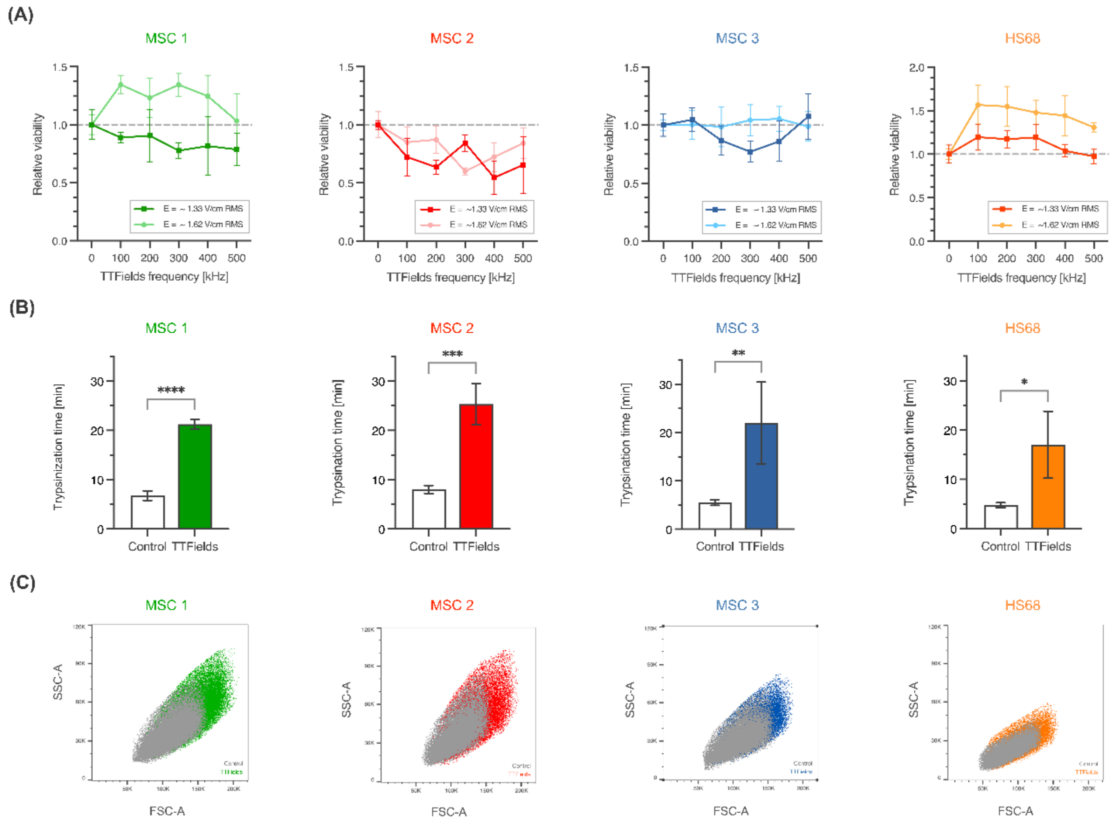

Supplement: Supplementary file 1 — Supplementary Material 1 [file 11060_2024_4740_MOESM1_ESM.pdf]
